# Clinician Attitudes towards Analgesia for Chronic Limb Threatening Ischaemia: A Survey of Vascular Specialists to Inform a Topical Morphine Trial

**DOI:** 10.1016/j.ejvsvf.2025.12.001

**Published:** 2025-12-31

**Authors:** Henry Davies, Marie-José Vleugels, Mimmie Kwong, Anahita Dua, Nasim Akhtar, Barend M.E. Mees, Sarah Mitchell, David Russell

**Affiliations:** a Leeds Institute of Cardiovascular and Metabolic Medicine, University of Leeds, Leeds, UK; b Department of Vascular Surgery, Maastricht UMC+, Maastricht, the Netherlands; c Department of Vascular Surgery, University of California, Davis, CA, USA; d Department of Vascular Surgery, University of Harvard, Cambridge, MA, USA; e Department of Vascular Surgery, Bradford Teaching Hospitals Foundation Trust, Bradford, UK; f Leeds Institute of Health Sciences, University of Leeds, Leeds, UK; g Leeds Vascular Institute, Leeds Teaching Hospitals NHS Trust, Leeds, UK; h Leeds Institute of Clinical Trials Research, University of Leeds, Leeds, UK

**Keywords:** Analgesics, Ischaemia, Morphine, Pain management, Peripheral arterial disease, Topical administration

## Abstract

**Objective:**

Current analgesic strategies often treat pain associated with chronic limb threatening ischaemia (CLTI) inadequately and frequently result in significant side effects. This cross sectional, web based, vascular specialist survey study aimed to explore clinician perspectives on current pain management and the potential role of topical morphine to inform future research in this area.

**Methods:**

A cross sectional online survey was distributed to vascular surgeons, physicians, and allied health professionals involved in the care of patients with CLTI. The 16 question survey included both closed and open ended items exploring perceptions of current analgesia adequacy, experience with topical morphine (e.g., morphine in gel preparations), and attitudes toward future clinical research. The questionnaire was disseminated via national and international vascular societies, professional mailing lists, and social media platforms. Responses were recorded anonymously and stratified using five point Likert scales (strongly disagree to strongly agree).

**Results:**

Of the 104 respondents, most were consultant vascular surgeons (57%) or speciality registrars (14%), with the majority based in the UK (89%). Pain control for CLTI was rated as suboptimal by 96% of respondents in outpatient settings and 78% in inpatient settings. No respondents strongly agreed that pain was adequately managed in either context. Only eight respondents reported experience using topical morphine; of these, three reported it as effective most of the time and five said it was effective sometimes. Nonetheless, 79% expressed willingness to randomise patients with ulcers to a trial of topical morphine, and 85% were willing to include patients without ulcers. All but one of the 104 respondents agreed that reducing systemic analgesia would be of significant patient benefit.

**Conclusion:**

There is both a clinical need and strong support among vascular clinicians for research into optimised analgesic regimens for CLTI. Despite limited current experience with topical morphine, the majority are willing to recruit to a trial.

## HIGHLIGHTS

• Vascular clinicians report poor pain control for chronic limb threatening ischaemia.
• Clinician experience with topical morphine is limited, although some report effectiveness.
• Many clinicians are willing to recruit patients to a future topical morphine trial.
• Findings show professional support for developing future studies on topical morphine and other pain strategies.

## INTRODUCTION

Chronic limb threatening ischaemia (CLTI), the end stage of peripheral artery disease, affects approximately 3 500 new cases per million individuals per year.^1^ Peripheral artery disease reduces blood flow to the legs due to arterial narrowing and progresses to CLTI when rest pain develops, with or without skin ulceration.^1^^,^^2^ Patients with CLTI experience severe pain that is difficult to manage with standard analgesics; this is partly due to the complex underlying mechanisms inherent to ischaemic pain.^3^ Despite there being a more comprehensive understanding of nociceptive and neuropathic pain pathways, the interplay between them and other pathways involved in ischaemic pain is considerably less well understood, especially in ischaemic pain caused by CLTI.^4^ The processes and pathways that culminate in the overall experience of pain in CLTI vary between patients and are influenced by factors such as tissue viability, anatomic location, local tissue composition, nerve ending integrity, and up or down regulation of pain receptors, which can be affected by the inflammatory state of the localised tissue, among other factors.^5^^,^^6^ Consequently, patients experience a varying mixture of nociceptive, neuropathic, metabolic, and inflammatory components contributing to their pain.^7^^,^^8^

In 1986, the World Health Organisation (WHO) developed and introduced the WHO pain ladder for the management of cancer pain.^9^ Since then, this analgesic protocol has been widely adopted internationally to treat all types of pain. However, subsequent evidence and experience suggest that it may be less effective for other, far more prevalent, causes of pain, and there is a lack of research on strategies to manage these conditions. For example, a 2017 systematic review of ischaemic pain in CLTI was unable to make any recommendations based on the reviewed papers.^10^

Topical morphine (oral morphine mixed in a gel applied directly to ulcers) presents a theoretically promising alternative, with small pilot studies and case series reporting local analgesic effects on painful leg ulcers via peripheral opioid receptors while avoiding systemic side effects.^11^, ^12^, ^13^ However, most ulcers included in these studies were non-arterial in nature and, to date, there is no high level evidence to support clinical use. To help inform the need for a future clinical trial on topical morphine for ischaemic pain in CLTI, a survey of vascular specialists was conducted. The objectives were to: assess current pain management practices in CLTI; determine the proportion of vascular clinicians currently using topical morphine and their perceptions of its efficacy; assess potential to recruit to trial; and identify other potential treatment arms for inclusion in the trial.

## METHODS

**Figure 1:**
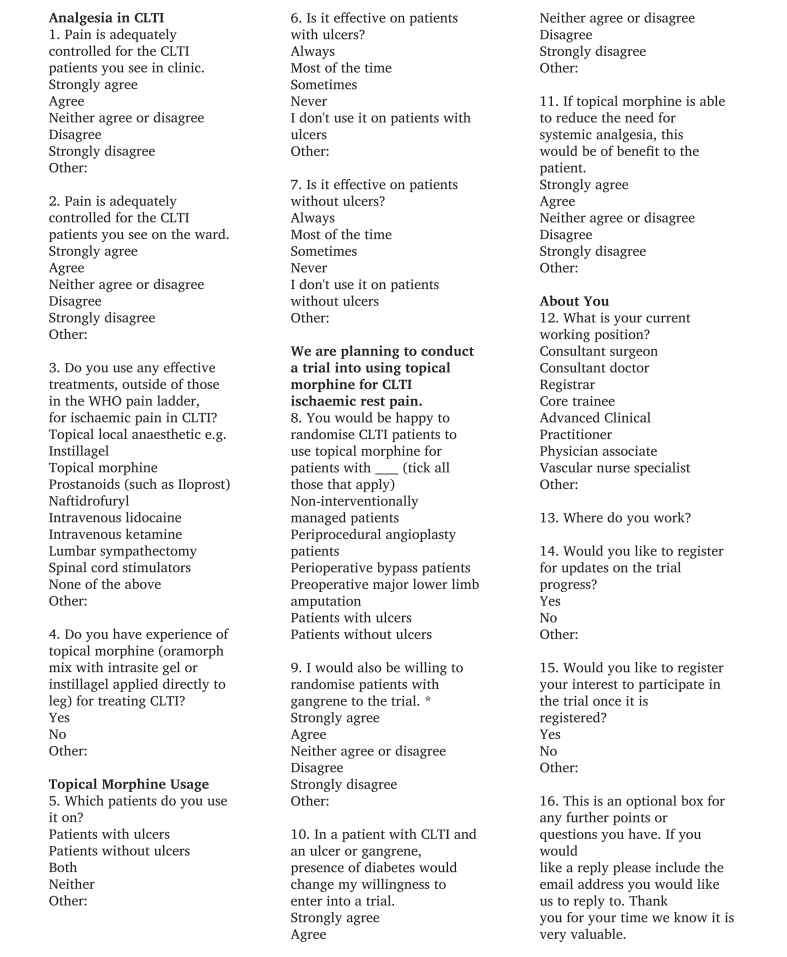
Full questionnaire used in the vascular specialist survey. This figure presents the complete 16 item web based survey administered to vascular surgeons, physicians, and allied health professionals managing chronic limb threatening ischaemia (CLTI). It includes all questions and response options on current analgesic practice, use of therapies outside the World Health Organisation (WHO) pain ladder, experience with topical morphine, willingness to randomise patients to a topical morphine trial and clinician demographics.

An online vascular specialist survey was designed using Google Forms (Google LLC, USA) and distributed between May and July 2025, targeting vascular surgeons, doctors, and allied health professionals managing patients with CLTI. The questionnaire was developed in consultation with vascular and palliative care professionals experienced in using topical morphine. It was piloted on ten local vascular surgeons to assess clarity and content relevance before wider dissemination. The survey included both closed and open ended questions. Fig. 1 shows the full questionnaire used in the survey. Domains covered included: perceived adequacy of current CLTI pain control; use of analgesics outside the WHO pain ladder; experience with topical morphine; trial acceptability and willingness to randomise pr-e, peri- and post-procedural, and non-operatively managed patients with CLTI; and willingness to include patients with ulcers, without ulcers, with diabetes, and with gangrene. Responses were collected anonymously, with optional registration for trial updates and participation. The built in settings of Google Forms restricted each respondent to a single submission, to prevent duplicate entries.

### Participant recruitment

Invitations were: sent to vascular societies (The Vascular Society of Great Britain and Northern Ireland; The Vascular and Endovascular Research Network, UK; The Rouleaux Club Vascular Trainees’ Association for Great Britain and Northern Ireland; The Netherlands Vascular Society); posted on LinkedIn and X; and directly emailed to vascular clinicians in the UK, Netherlands, and USA through professional societal mailing lists and networks. Participating countries reflected the distribution of active professional society networks and existing collaborative links among the study investigators.

### Analysis

Quantitative data were analysed using R software (version 4.1.2; R Foundation for Statistical Computing, Vienna, Austria). Descriptive statistics were used to summarise responses.

Ethics approval was not required for this study, in accordance with national and or local guidelines. Participation was voluntary and informed consent was obtained from all respondents.

The study was reported in accordance with the CROSS (Consensus Based Checklist for Reporting of Survey Studies) guidelines.^14^

## RESULTS

### Respondents

A total of 104 responses were received from a broad range of vascular centres, predominantly in the UK. Geographical distribution was: UK: 93 (89%); Europe: eight (8%); USA: three (3%). Most respondents were consultant vascular surgeons (59%), followed by speciality registrars (14%) and consultant physicians (14%). Other respondents included vascular nurse specialists, advanced clinical practitioners, physician assistants, core trainees, and podiatrists.

### Assessment of current pain management

**Outpatient.** Pain control for CLTI was rated as suboptimal by 96% of respondents, including 60.2% who disagreed and 22.3% who strongly disagreed that pain was adequately controlled.

**Inpatient.** Pain control for CLTI was rated as suboptimal by 96% of respondents, including 60.2% who disagreed and 22.3% who strongly disagreed that pain was adequately controlled.

**Figure 2:**
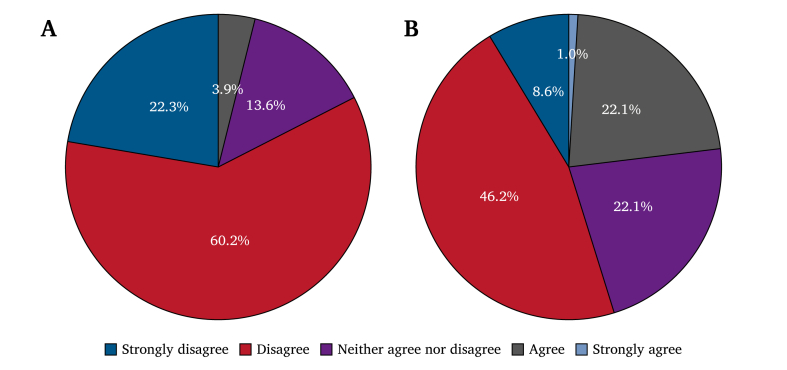
Vascular specialist ratings of pain control adequacy for patients with chronic limb threatening ischaemia (CLTI). **(**A) Outpatient responses to the statement “Pain is adequately controlled for the CLTI patients you see in clinic” (*n* = 103). **(**B) Inpatient responses to the statement “Pain is adequately controlled for the CLTI patients you see on the ward” (*n* = 104).

Vascular specialists’ ratings of pain control adequacy are presented in Fig. 2A for outpatient settings and in Fig. 2B for inpatient settings.

### Alternative therapies

Seventy six percent of respondents reported using one or more interventions outside the WHO pain ladder: iloprost or prostanoids 27%; lumbar sympathectomy 19%; topical anaesthetic agents (e.g., Instillagel) 10%; intravenous ketamine 7%; spinal cord stimulators 5%; and intravenous lidocaine 2%.

### Topical morphine experience

Eight respondents had experience of using topical morphine on CLTI patients with ulcers. Of these, three reported that it was effective most of the time, and five reported that it was effective sometimes. Of the four respondents who had experience of using it on patients without ulcers, three said it was effective sometimes and one said it was effective most of the time, indicating good efficacy but a very small sample size.

### Recruitment feasibility

**Patients with ulcers.** Eighty two respondents (78.8%) were willing to randomise non-interventionally managed patients; 73 (70.2%) were willing to randomise peri-procedural patients; 65 (62.5%) were willing to randomise peri-operative surgical bypass patients; and 71 (68.3%) were willing to randomise peri-operative amputation patients.

**Patients without ulcers.** Eighty eight respondents (84.6%) were willing to randomise non-interventionally managed patients; 80 (76.9%) were willing to randomise peri-procedural patients; 72 (69.2%) were willing to randomise peri-operative surgical bypass patients; and 74 (71.2%) were willing to randomise peri-operative amputation patients.

**Gangrene and diabetes.** Seventy four respondents (73%) would include patients with gangrene. The presence of diabetes did not substantially affect willingness to enrol.

### Value proposition

Ninety seven respondents agreed or strongly agreed (95%) that reducing systemic analgesia would be of benefit to patients. Several respondents commented on the need for improved analgesic options, particularly in palliative scenarios.

## DISCUSSION

This survey highlights a significant unmet need in ischaemic pain management for CLTI. The reported dissatisfaction with current analgesia, especially among experienced consultants, underscores the importance of developing new strategies. Although experience with topical morphine is limited, a proportion of respondents expressed interest in its further investigation.

The findings indicate that a multicentre randomised trial could be acceptable to vascular clinicians, with many expressing willingness to participate and randomise across relevant patient subgroups. Responses regarding willingness to include peri-operative patients, those with and without ulcers, and those with gangrene will help guide the design of future studies. Concerns about diabetes or gangrene as exclusion factors were minimal, suggesting a broad potential recruitment base.

The 2020 ESVS Clinical Practice Guidelines on the Management of Chronic Limb Threatening Ischaemia recommend urgent referral, comprehensive vascular assessment, and revascularisation where feasible, but acknowledge the absence of high level evidence for effective analgesic strategies in non-reconstructable disease.^1^ Current pain control approaches are not specifically addressed beyond general palliative principles, underscoring the need for targeted research into symptom relief for this population. This study contributes to this field by providing insights into clinician perspectives on current practice and the appetite for future trials evaluating topical morphine in CLTI pain management.

The management of ischaemic pain in CLTI remains challenging due to the mixed nociceptive and neuropathic components, limited efficacy of standard opioid and non-opioid regimens, and the frequent presence of comorbidities that limit systemic drug use. In the interim, a multidisciplinary approach combining optimised pharmacological therapy, psychological support, and early palliative involvement may help mitigate suffering while awaiting stronger evidence.

Building on the results of this survey, a future pragmatic, multicentre, randomised controlled trial could compare topical morphine (morphine solution in gel applied directly to ulcers) with standard analgesic care in patients with non-reconstructable CLTI and ischaemic ulceration. Primary outcomes should include pain intensity and patient reported quality of life, with secondary outcomes assessing systemic opioid use, wound healing, and adverse events. Parallel qualitative work could explore acceptability among patients and staff to refine trial implementation and interpretability.

Limitations of this study included the potential selection bias of participants, as respondents were self-selected and may have been more interested in analgesia research than the wider vascular clinician community. The sample was also not fully representative geographically or by professional role, with UK based consultants forming most respondents. The accuracy of the findings may have been affected by recall bias, as participants reported their practices and experiences from memory. Willingness to participate in a trial was assessed in a hypothetical context and does not account for patient and carer perspectives, resource availability, or ethical and logistic factors that would influence real world implementation. Finally, the survey format limited the depth of exploration into clinicians’ reasoning, which could be better addressed through qualitative interviews or focus groups.

Patient involvement will form a key part of the next phase of this research. Patient and public representatives will be engaged in the design and prioritisation of outcomes for the proposed randomised controlled trial of topical morphine, ensuring that study objectives and endpoints align with patient needs and values.

Taken together, these findings highlight a clear need for prospective clinical research to evaluate effective pain management strategies for CLTI. The next step should be a feasibility study to prepare for a pragmatic randomised controlled trial comparing standard analgesic care with standard analgesic care plus topical morphine. Future studies should incorporate patient reported outcomes and qualitative assessments to better understand treatment acceptability and impact on quality of life.

From a clinical perspective, these findings highlight a clear need to strengthen analgesic strategies for patients with CLTI, particularly where current therapies provide limited benefit. While this survey does not itself define changes to practice, it confirms that vascular specialists recognise the gap in effective pain management and are willing to support further evaluation of topical morphine. These results therefore directly inform the next stage of research, namely a feasibility study followed by a pragmatic randomised controlled trial of topical morphine in CLTI related ischaemic pain. Such a trial would provide the evidence required to determine whether topical morphine could be incorporated into future clinical pathways.

### Conclusion

Vascular specialists managing CLTI report suboptimal pain control and support further investigation into novel analgesic approaches. Topical morphine is viewed by some as a promising option, and many vascular specialists would consider recruiting to a randomised controlled trial of its use. A future trial designed around current pain management practice could provide valuable evidence to guide clinical care and improve patient outcomes. These findings offer insight into vascular specialist perspectives and highlight the need for coordinated research efforts to advance patient care in CLTI.

## FUNDING

This study did not receive funding

## USE OF GENERATIVE AI TOOLS

During the preparation of this work the author(s) used ChatGPT (OpenAI*)* in order to assist with proof reading and formatting. After using this tool, the author(s) reviewed and edited the content as needed and take(s) full responsibility for the content of the publication.

## CONFLICT OF INTEREST

All authors declare no conflicts of interest.
